# The Impact of Superoxide Dismutase-1 Genetic Variation on Cardiovascular and All-Cause Mortality in a Prospective Cohort Study: The Yamagata (Takahata) Study

**DOI:** 10.1371/journal.pone.0164732

**Published:** 2016-10-18

**Authors:** Yoichiro Otaki, Tetsu Watanabe, Satoshi Nishiyama, Hiroki Takahashi, Takanori Arimoto, Tetsuro Shishido, Takuya Miyamoto, Tsuneo Konta, Yoko Shibata, Hidenori Sato, Ryo Kawasaki, Makoto Daimon, Yoshiyuki Ueno, Takeo Kato, Takamasa Kayama, Isao Kubota

**Affiliations:** 1 Department of Cardiology, Pulmonology, and Nephrology, Yamagata University School of Medicine, Yamagata, Japan; 2 Genome Informatics Unit, Institute for Promotion of Medical Science Research, Faculty of Medicine, Yamagata University, Yamagata, Japan; 3 Department of Public Health, Graduate School of Medical Science, Yamagata University, Yamagata, Japan; 4 Department of Endocrinology and Metabolism, Hirosaki University Graduate School of Medicine, Aomori, Japan; 5 Global Center of Excellence Program Study Group, Yamagata University School of Medicine; Yamagata, Japan; Kurume University School of Medicine, JAPAN

## Abstract

**Background:**

Oxidative stress is a major cause of cardiovascular disease. Superoxide dismutase-1 (SOD1) is an antioxidant that protects against oxidative stress. Deoxyribonucleic acid (DNA) variations such as single nucleotide polymorphism (SNP) or haplotypes within the SOD gene are reportedly associated with the development of cardiovascular disease. However, it remains to be determined whether SOD1 variability is associated with cardiovascular or all-cause mortality in the general population.

**Methods and Results:**

This prospective cohort study included 2799 subjects who participated in a community-based health study with a 10-year follow-up. We genotyped 639 SNPs and found the association of SNP rs1041740 and rs17880487 within a SOD1 gene with cardiovascular mortality. There were 193 deaths during the follow-up period including 57 cardiovascular deaths. Multivariate Cox proportional hazard regression analysis revealed that the homozygous T-allele of rs1041740 was associated with all-cause and cardiovascular deaths after adjusting for confounding factors. The net reclassification index was significantly improved by adding rs1041740 as a cardiovascular risk factor. On the other hand, cardiovascular death was not observed in homozygous T-allele carriers of rs17880487. Haplotype analysis identified the haplotype with T-allele of rs1041740 and that with T-allele of rs17880487 as increasing and decreasing susceptibility for cardiovascular mortality, and it had complementary SNP sequences.

**Conclusion:**

Variation in the SOD1 gene was associated with cardiovascular deaths in the general population.

## Introduction

Despite advances in medicine, cardiovascular disease remains a major public health problem associated with high mortality [1,2]. The development of cardiovascular disease is associated with multiple genetic and cardiovascular risk factors. Single nucleotide polymorphism (SNP) is the most frequent type of human population deoxyribonucleic acid (DNA) variation and haplotype is defined as a combination of SNP alleles along a chromosome [3,4]. Whether DNA variation can be used to identify genes that increase the risk of cardiovascular disease is currently under discussion [5].

Oxidative stress is the excessive accumulation of reactive oxygen species (ROS) relative to antioxidant activity and is a major cause of cardiovascular disease [6,7]. Superoxide dismutase-1 (SOD1) is an antioxidant protein that plays a pivotal role in reducing ROS by catalysing superoxide into oxygen and hydrogen peroxide [8]. Previous reports demonstrated that some *SOD1* SNPs are associated with the development of cardiovascular disease [9,10,11].

The present study examined whether *SOD1* DNA variation can predict all-cause and cardiovascular mortality in the general population.

## Methods

### Ethics statement and study population

The institutional ethics committee of Yamagata University School of Medicine approved the study, and all participants provided written informed consent. The procedures were performed in accordance with the Helsinki Declaration.

Our analysis was part of a community-based health study of inhabitants in the town of Takahata in northern Japan (total population 26,026). Community members, aged >40 years were invited to participate in this study. In 2004 and 2005, 2,968 subjects (1,343 males and 1,625 females) were enrolled in the study but 169 subjects were excluded due to their incomplete data and study withdrawal.

### Genotyping

Genotyping was performed with the Invader assay (Third Wave Technologies, Madison, WI, USA) and Taq Man Allelic discrimination assay. Reagents were purchased from Applied Biosystems (Foster City, CA, USA). Taq Man probes that can distinguish SNPs after polymerase chain reaction (PCR) were designed and synthesized by Applied Biosystems. One allelic probe was labelled with the fluorescent FAM dye, and the other with the fluorescent VIC dye. PCR was performed with the Taq Man Universal Master Mix with primers at concentrations of 225 nM and Taq Man MGB probes at concentrations of 50 nM. Reactions were performed in 382 well plates in a total volume of 3 μL using 3.0 ng genomic DNA. The plates were then placed in a GeneAmp PCR system 9700 (Applied Biosystems) and heated at 95°C for 10 min, followed by 40 cycles at 92°C for 15 sec and at 60°C for 1 min, with a final incubation at 25°C. The fluorescent intensities of each well in the plates were then read by the Prism 7900HT instrument (Applied Biosystems). Fluorescent data files from each plate were analyzed with the SDS 2.0 allele calling software (Applied Biosystems). Several data points were eliminated to preserve the reliability of the assay system (missing data due to poor signal intensity < 1.1%) [12].

### Haplotype statistics

Haplotype was identified according to the expectation maximization (EM) algorithm. Haplotypes with extremely low frequency were excluded from haplo.score analysis using R software. To examine the association of haplotype with cardiovascular mortality, haplo.scores were calculated. All haplotype analyses were performed with the haplo.stats plug in.

### Definition of cardiovascular risks

Hypertension was defined as systolic blood pressure ≥140 mmHg, diastolic blood pressure ≥90 mmHg, or antihypertensive medication use. Diabetes mellitus (DM) was defined as having fasting blood glucose ≥126 mg/dL, glycosylated hemoglobin A1c ≥6.5% (National Glyco hemoglobin Standardization Program), or anti-diabetic medication use. Hyperlipidemia was defined as total cholesterol ≥220 mg/dL, triglyceride ≥150 mg/dL, or anti-hyperlipidemic medicine use. Family histories of cardiovascular disease and previous cardiovascular disease were determined by self-reported questionnaires. Framingham risk score was calculated according to previous report [13].

### Biochemical markers

Blood samples were obtained to measure brain natriuretic peptide (BNP). These samples were transferred to chilled tubes containing 4.5 mg ethylene diamine tetra acetic acid disodium salt and aprotinin (500 U/mL), and centrifuged at 1,000 *g* for 15 minutes at 4°C. The clarified plasma samples were frozen, stored at -70°C, and thawed just before assay. BNP concentrations were measured using a commercially available radioimmunoassay specific for human BNP (Shiono RIA BNP assay kit, Shionogi Co. Ltd., Tokyo, Japan) [14].

Estimated glomerular filtration rate (eGFR) was calculated using the modification of diet in renal disease equation with the Japanese coefficient [15].

Total cholesterol, triglyceride, high density lipoprotein cholesterol, fasting blood glucose, and glycosylated hemoglobin A1c were also measured at the same time.

### Endpoint and follow-up

All subjects were prospectively followed for a median period of 3,397 days (interquartile range, 3,097–3,443 days). The endpoint was all-cause death, which was further divided into cardiovascular and non-cardiovascular death. Cardiovascular death was defined as death due to coronary artery disease, heart failure, arrhythmia, stroke, or aortic artery disease. The cause of death was determined by reviewing death certificates through the end of 2014. The death code (International Classification of Diseases, 10^th^ Revision) and place of death were reviewed.

### Statistical analysis

Continuous variable normality was checked with a Kolmogorov-Smirnov-Lillefors test. Because BNP was not normally distributed, we used log_e_ BNP for all analyses. All values are expressed as the mean ± standard deviation. Continuous and categorical variables were compared with t-tests and chi-square tests, respectively. Differences among *SOD1*genetic variations were assessed with analysis of variance (ANOVA) with Bonferroni post hoc tests. A Cox proportional hazard analysis was performed to determine independent predictors for all-cause deaths, cardiovascular deaths, and non-cardiovascular deaths, and cardiovascular risk factors were entered into the multivariate analysis. We calculated the net reclassification index (NRI) and integrated discrimination index (IDI) to measure the quality of improvement for the correct reclassification according to the addition of SNPs within *SOD1* to the multivariate model. Survival curves were constructed with the Kaplan-Meier method and compared using log-rank tests. P < 0.05 was considered statistically significant. All statistical analyses were performed with standard statistical program packages (JMP version 11; SAS Institute Inc., Cary, NC, USA and R 3.0.2 with additional packages including Rcmdr, Epi, pROC, and PredictABEL).

## Results

### Baseline characteristics and clinical characteristics related SNP rs1041740

**Fig 1:**
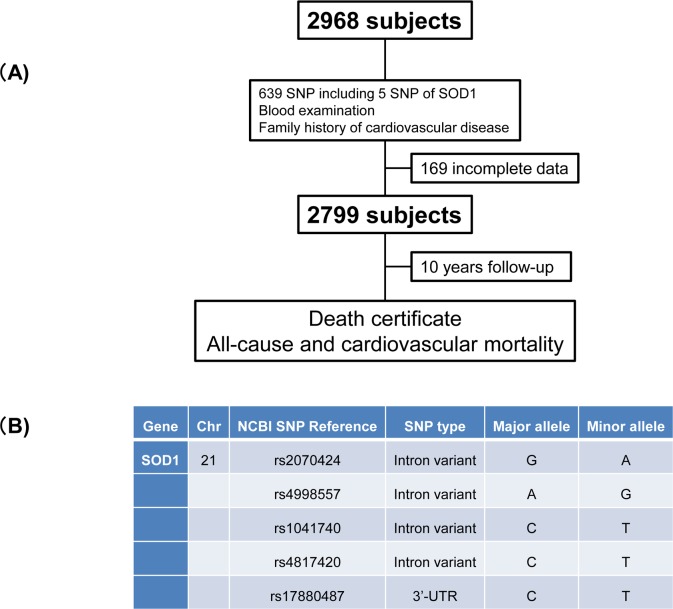
The study protocol (A) and polymorphism in *SOD1* gene examined in the study (B). Chr, chromosome; SNP, single nucleotide polymorphism; SOD, superoxide dismutase; UTR, untranslated region.

The Yamagata study protocol and the genotyped SNPs within *SOD1* are shown in Fig 1. During the follow-up period, there were 193 all-cause deaths including 57 cardiovascular and 136 non-cardiovascular deaths. We found the association of *SOD1* SNP with cardiovascular mortality using in silico analysis from the 639 SNPs examined in this study. The homozygous T-allele (TT), heterozygous (TC), and homozygous C-allele (CC) carriers of rs1041740 were identified in 307 (11%), 1,259 (45%), and 1,233 (44%) subjects, respectively. Also, TT, TC, and CC carriers of rs17880487 were identified in 29 (1%), 465 (17%), and 2,305 (82%), respectively. As shown in Table 1, rs1041740 TT carriers had a higher BNP level compared to other groups. Moreover, rs1041740 TT carriers had a greater family history of cardiovascular disease compared to the rs1041740 CC carriers. There were no significant differences in age, gender, or prevalence rates of previous cardiovascular disease, smoking, hypertension, DM, or hyperlipidemia among rs1041740 genotypes. The rs1041740 TT carriers did not overlap the rs17880487 TT carriers.

**Table 1:** Baseline and clinical characteristics among rs1041740 genotypes.

Variables	All subject n = 2799	Homozygous C-allele carriers n = 1233	Heterozygous carriers n = 1259	Homozygous T-allele carriers n = 307
**Age, years**	63 ± 10	63 ± 10	63 ± 10	64 ± 10
**Male/female, n**	1270/1529	556/677	562/697	152/155
**Family history of cardiovascular disease, n (%)**	476 (17%)	178 (14%)	241 (19%)	57 (19%)#
**Previous CVD, n (%)**	383 (14%)	149 (12%)	187 (15%)	47 (15%)
**Previous cancer, n (%)**	55 (2.0%)	23 (1.8%)	27 (2.1%)	5 (1.6%)
**Smoking, n (%)**	906 (32%)	398 (32%)	400 (32%)	108 (35%)
**Hypertension, n (%)**	1038 (37%)	446 (36%)	477 (38%)	115 (37%)
**Diabetes mellitus, n (%)**	191 (7%)	92 (7.5%)	82 (6.5%)	17 (5.5%)
**Hyperlipidemia, n (%)**	1044 (37%)	479 (39)	454 (36)	111 (36)
** Systolic BP, mmHg**	134 ± 16	134 ± 16	134 ± 16	135 ± 16
** Diastolic BP, mmHg**	79 ± 10	79 ± 10	79 ± 10	79 ± 10
** HbA1c, %**	5.6 ± 0.7	5.7 ± 0.7	5.6 ± 0.7	5.6 ± 0.5
** FBG, mg/dL**	94 ± 17	95 ± 18	93 ± 16	94 ± 16
** TC, mg/dL**	201 ± 31	201 ± 32	200 ± 31	200 ± 32
** HDLc, mg/dL**	59 ± 14	59 ± 14	59 ± 14	60 ± 15
** TG, mg/dL**	106 ± 64	107 ± 62	104 ± 65	107 ± 66
** eGFR, mL/min/1.73 m**^**2**^	81 ± 16	82 ± 16	80 ± 16	83 ± 18^†^
** Log**_**e**_ **BNP, pg/mL**	3.02 ± 0.83	3.00 ± 0.83	3.00 ± 0.83	3.14 ± 0.88*^†^
**Framingham risk score**	14 ± 4	14 ± 4	14 ± 4	14 ± 4
**SNP SOD gene**				
** rs2070424, AA/AG/GG**	644/1433/722	58/454/721	279/979/1	307/0/0
** rs4998557, AA/AG/GG**	720/1433/646	720/454/59	0/979/280	0/0/307
** rs1041740, CC/CT/TT**	1233/1259/307	1233/0/0	0/1259/0	0/0/307
** rs4817420, CC/CT/TT**	1233/1259/307	1233/0/0	0/1259/0	0/0/307
** rs17880487, CC/CT/TT**	2305/465/29	911/293/29	1087/172/0	307/0/0

Data are expressed as mean ± standard deviation or number (%)

BNP, brain natriuretic peptide; BP, blood pressure; CVD, cardiovascular disease; eGFR, estimated glomerular filtration rate; FBG, fasting blood glucose; HbA1c, glycosylated hemoglobin A1c; HDLc, high density lipoprotein cholesterol; SNP, single nucleotide polymorphism; SOD, superoxide dismutase; TC, total cholesterol; TG, triglyceride.

*p<0.05 vs. homozygous C-allele carriers

^†^p<0.05 vs. heterozygous carriers by analysis of variance (ANOVA) with Bonferroni test.

^#^p < 0.05 by chi-square test.

Clinical characteristics related to rs17880487 were shown in S1 Text. Interestingly, the rs17880487 TT carriers had a lower prevalence of family history of cardiovascular disease compared to rs17880487 TC and CC carriers. Cardiovascular death was not observed in rs17880487 TT carriers. The impact of rs1041740 TT carriers and rs17880487 TT carriers on the cardiovascular disease was shown in S1 Fig.

### All-cause and cardiovascular mortality and SNP rs1041740

**Fig 2:**
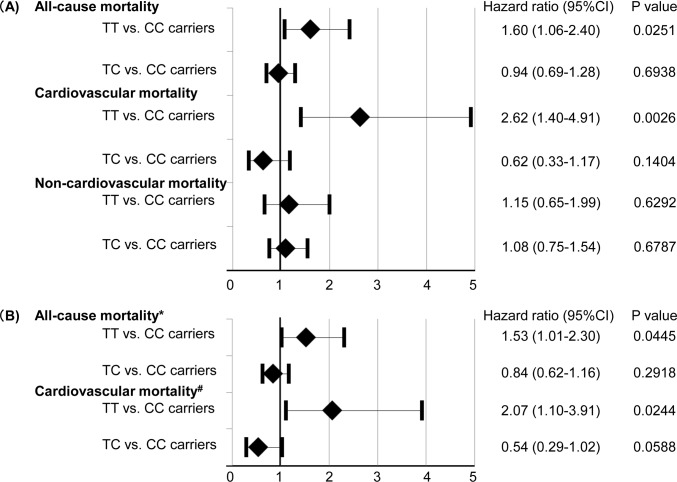
Univariate (A) and multivariate (B) Cox proportional hazard regression analyses for mortality. *after adjustment for age, gender, family history of cardiovascular disease, previous cardiovascular disease, smoking, hypertension, diabetes mellitus, hyperlipidemia, total cholesterol, high density lipoprotein cholesterol, systolic blood pressure, BNP, and eGFR. ^#^after adjustment for age, gender, BNP, eGFR, and Framingham risk score. BNP, brain natriuretic peptide; CI, confidence interval; eGFR, estimated glomerular filtration rate.

As shown in Fig 2A, univariate Cox proportional hazard regression analysis demonstrated that the homozygous T-allele of rs1041740 was related to all-cause deaths and cardiovascular deaths in the general population. On the other hand, it was not related to non-cardiovascular deaths. To determine the risk factors associated with all-cause deaths, cardiovascular deaths, and non-cardiovascular deaths, cardiovascular risk factors were entered into the multivariate Cox proportional hazard model. It demonstrated that the homozygous T-allele of rs1041740 was an independent predictor of future all-cause and cardiovascular mortality (all-cause deaths, adjusted hazard ratio, 1.53; 95% confidence interval, 1.01–2.30; P = 0.0445 and cardiovascular deaths, adjusted hazard ratio, 2.07; 95% confidence interval, 1.10–3.91; P = 0.0244; Fig 2B).

**Fig 3:**
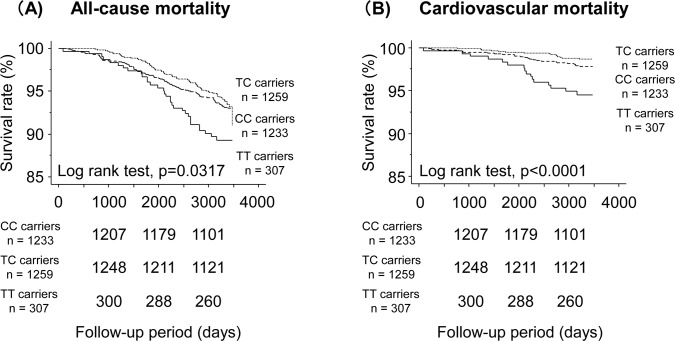
Kaplan-Meier analysis of all-cause deaths **(A)** and cardiovascular deaths **(B)** among rs1041740 genotype.

Kaplan-Meier analysis demonstrated that rs1041740 TT carriers had a higher rate of all-cause and cardiovascular mortality compared to rs1041740 TC and CC carriers (Fig 3).

### Subgroup analysis in subjects without previous cardiovascular disease

**Fig 4:**
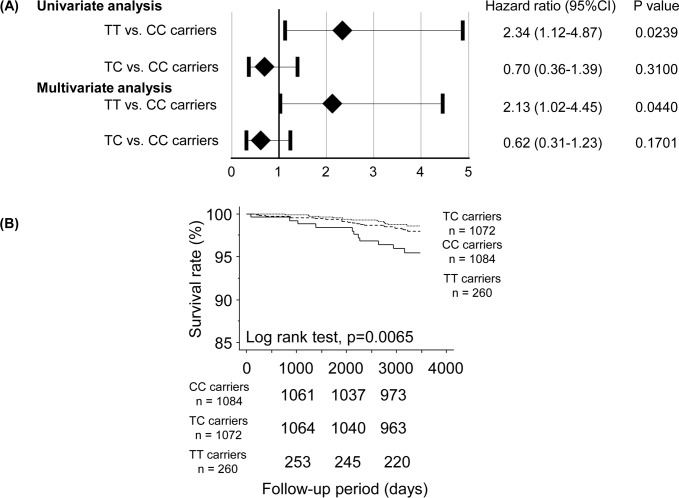
(A) Multivariate Cox proportional hazard regression analyses for cardiovascular mortality in subjects without previous cardiovascular disease. After adjustment for age, gender, and Framingham risk score. CI, confidence interval. (B) Kaplan-Meier analysis of cardiovascular deaths among rs1041740 genotype in subjects without previous cardiovascular disease.

Next, we performed subgroup analysis in subjects without previous cardiovascular disease to examine whether homozygous T-allele of rs1041740 could also predict cardiovascular mortality in these populations. As shown in Fig 4A, univariate Cox proportional hazard regression analysis demonstrated that the homozygous T-allele of rs1041740 was related to cardiovascular deaths in the general population. To determine the risk factors associated with cardiovascular deaths, age, gender, and Framingham risk score were entered into the multivariate Cox proportional hazard model. It demonstrated that the homozygous T-allele of rs1041740 was an independent predictor of cardiovascular mortality (adjusted hazard ratio, 2.13; 95% confidence interval, 1.02–4.45; P = 0.0440; Fig 4A).

Kaplan-Meier analysis demonstrated that rs1041740 TT carriers had a higher rate of cardiovascular mortality compared to rs1041740 TC and CC carriers (Fig 4B).

### Improvement of reclassification by adding SOD1 SNP rs1041740 to predict all-cause and cardiovascular mortality

To examine whether model fit and discrimination improve when the *SOD1* SNP rs1041740 was added to the known predictors of age, gender, family history of cardiovascular disease, previous cardiovascular disease, smoking, DM, eGFR, BNP, and Framingham risk score, we evaluated improvements in the NRI and IDI. As shown in Table 2, NRI and IDI to predict cardiovascular mortality was significantly improved by adding the *SOD1* SNP rs1041740 (Table 2).

**Table 2:** Statistics for model fit and improvement with the addition of rs1041740 on the prediction of cardiovascular mortality.

	NRI (95%CI, P value)	IDI (95%CI, P value)
Baseline model	Reference	Reference
**+rs1041740**	0.1084 (0.0101–0.2068, P = 0.0308)	0.0151 (0.0001–0.0301, P = 0.0492)

Baseline model includes age, gender, family history of cardiovascular disease, previous cardiovascular disease, smoking, diabetes mellitus, estimated glomerular filtration rate, brain natriuretic peptide, and Framingham risk score. 95%CI, 95% confidence interval; NRI, net reclassification index; IDI, integrated discrimination index.

### SOD1 haplotype and cardiovascular mortality

As shown in Table 3, nine haplotypes were identified in the study population. The sequence in haplotype 2 was GATTC, including the minor T-allele of rs1041740. The sequence in haplotype 4 was AGCCT, including the minor T-allele of rs17880487, indicating that these haplotypes had complementary SNP sequences. To examine whether SOD1 haplotype is related to cardiovascular mortality, the haplo.score was calculated, and the results suggested that haplotypes 2 and 4 confer susceptibility and protection for cardiovascular mortality, respectively (Haplotype 2 haplo.score, 1.9912, P = 0.0465; haplotype 4 haplo.score, -2.13818, P = 0.0325).

**Table 3:** Haplotype sequence, frequency, and hap score in SOD1 haplotypes.

Haplotype	rs4998557	rs2070424	rs1041740	rs4817420	rs17880487	Frequency	Hap score	P value
**1**	A	G	C	C	C	0.41956	-0.35348	0.7237
**2**	G	A	T	T	C	0.33439	1.9912	0.0465
**3**	G	A	C	C	C	0.15132	-0.33304	0.7391
**4**	A	G	C	C	T	0.09330	-2.13818	0.0325
**5**	G	G	C	C	C	0.00088		
**6**	A	A	C	C	C	0.00023		
**7**	G	G	T	T	C	0.00020	-0.31975	0.7492
**8**	A	A	C	C	T	0.00013		
**9**	G	G	C	C	T	0		

SOD, superoxide dismutase.

## Discussion

### Main findings

The results of this study revealed that the rs1041740 TT carriers in the *SOD1* gene had a higher level of BNP and a more frequent family history of cardiovascular disease compared to TC and CC carriers. Subjects with the rs1041740 TT genotype were at higher risk of all-cause and cardiovascular mortality; these associations were significant even after adjustments in a multivariate model with other known risk factors. Adding the rs1041740 TT genotype with known risk factors to the model enhanced the predictive capacity of cardiovascular mortality as NRI and IDI were significantly improved. We also identified protective and susceptible *SOD1* haplotypes for cardiovascular mortality.

### Family history of cardiovascular mortality and DNA variation of SOD1

A family history of cardiovascular disease is considered to be a risk factor for cardiovascular disease since it was reported to be closely associated with the development of cardiovascular disease [16,17]. Since inherited DNA sequence variants were suggested to play a causal role in cardiovascular disease susceptibility, SNP has been noted to explain the inherited cardiovascular disease [18]. In the present study, we showed that rs1041740 TT carriers had a higher prevalence of family history compared with rs1041740 CC carriers. On the other hand, rs17880487 TT carriers had no family history of cardiovascular disease. Although family history examined by self-reported questionnaires often includes recall bias, these results raised the possibility that DNA variation of *SOD1* contribute to cardiovascular disease heritability.

### BNP and rs1041740

BNP is a diagnostic marker for heart failure, that is constitutively synthesized and secreted from cardiomyocytes [19]. BNP levels are reportedly affected by several factors such as aging, gender, body mass index, and kidney function [20,21,22,23]. In the present study, the homozygous T-allele of rs1041740 carriers had a higher level of BNP compared to other groups despite similar levels of these factors. BNP expression is also reported to be increased in the several conditions such as left ventricular mechanical stretch, inflammation, and oxidative stress [24], suggesting that DNA variation of SOD1 modulated BNP expression in the general population. Previous studies have reported that BNP is a useful indicator of all-cause and cardiovascular deaths in the general population [25]. The results described here supported our hypothesis that genetic variation of *SOD1* is a risk for cardiovascular mortality.

### DNA variation of *SOD1* and mortality

No prospective study has examined the impact of *SOD1* DNA variation on all-cause and cardiovascular mortality in the general population. Previous reports concluded that the minor T-allele of rs1041740 is a risk for future cardiovascular mortality in patients with DM [26,27]. Similarly, we showed that the allele variation in *SOD1* is associated with all-cause and cardiovascular mortality in the general population. Although the SNPs examined in the present study were located in the intron or 3’-untranslated region, haplotype analysis showed that there were susceptible and protective haplotypes for cardiovascular mortality and they had complementary SNP sequences. Previous reports raised the possibility that DNA variation in these regions regulates gene expression by influencing transcription and translation processes [28,29]. Experimental studies have demonstrated that SOD1 works as an antioxidant and protects cardiomyocytes from oxidative stress in a myocardial infarction model [30,31]. Interestingly, SNP within *SOD1* was the independent predictor of cardiovascular deaths after adjustment for cardiovascular risk factors. The genetic variation of *SOD1* may deteriorate cardiovascular mortality through reduced SOD1 expression and activity with resultant increase in oxidative stress. Considering the role of SOD1, it is possible that variation in the gene encoding this protein is related to cardiovascular disease hereditability.

### Limitation

The strengths of this study are that it was conducted with a large number of participants and had a long follow-up period. However, there are some limitations. First, this study collected baseline information at a single time point. Subsequent medical interventions may have affected serum BNP levels. Second, non-fatal diseases were not assessed, which could result in an underestimation of the association between SNPs within *SOD1* and clinical outcomes. Third, a missense mutation of *SOD1* is associated with the development of amyotrophic lateral sclerosis [32]. Taking the role of SNPs used in the present study into account, it is unlikely that these SNPs induce amyotrophic lateral sclerosis. Finally, protein expression and activity of SOD1 and oxidative stress markers were not measured.

## Conclusions

*SOD1* variation was found to be associated with cardiovascular mortality in the general population. This knowledge may improve re-classification beyond known risk factors and underlies the importance of cardiovascular disease hereditability.

## Supporting Information

S1 Fig The association between DNA variation and family history of cardiac disease (A) and cardiovascular deaths (B). TT, homozygous T-allele carriers; TC, heterozygous carriers; CC, homozygous C-allele carriers.
(TIF)

S1 Text Clinical characteristics related SNP rs17880487. (DOCX)
