# Supplementary figures and images for: The Impact of Superoxide Dismutase-1 Genetic Variation on Cardiovascular and All-Cause Mortality in a Prospective Cohort Study: The Yamagata (Takahata) Study
Source: PLoS One. 2016 Oct 18;11(10):e0164732. doi: 10.1371/journal.pone.0164732 (PMC5068777; doi:10.1371/journal.pone.0164732)

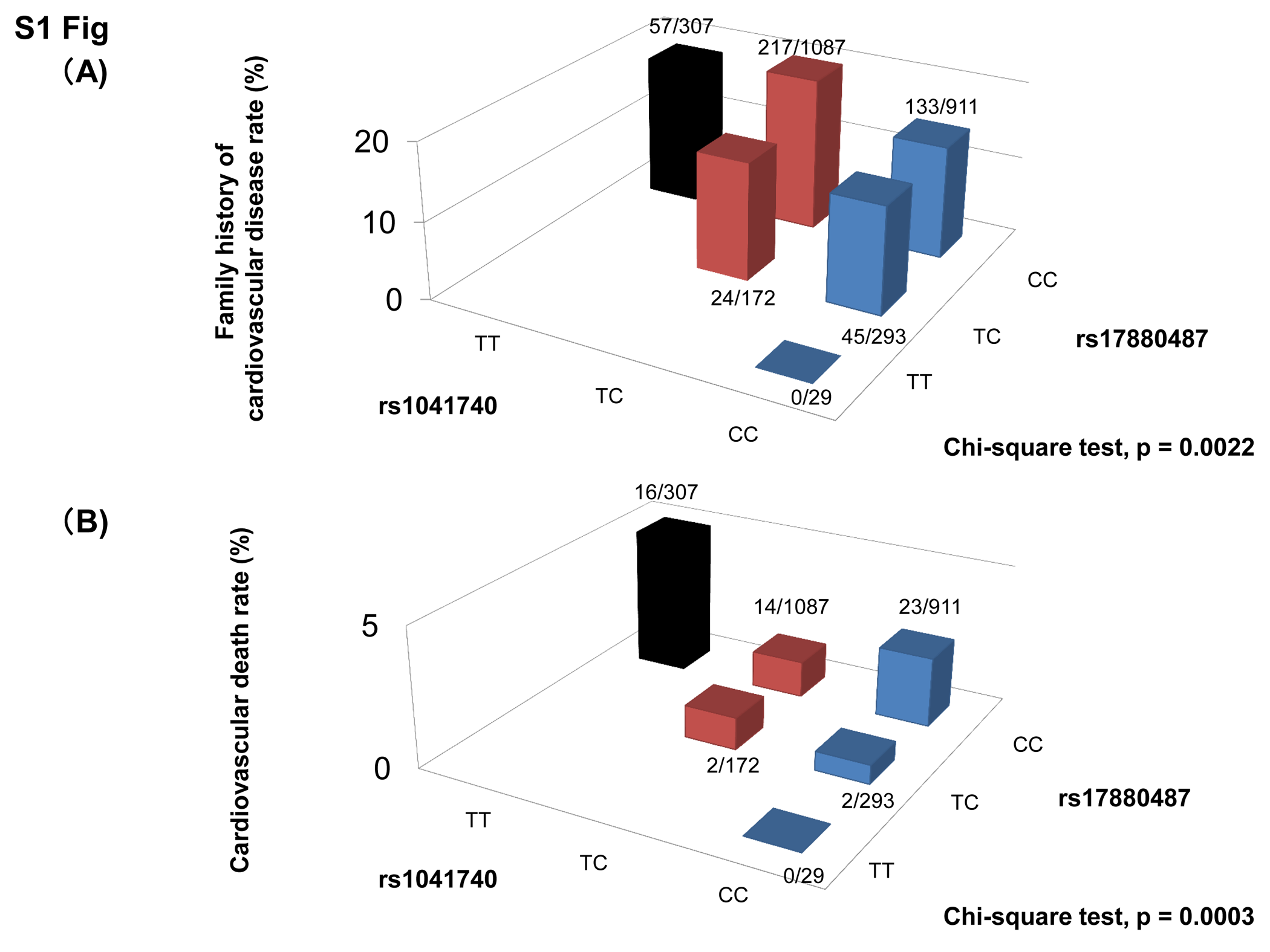

Supplement: S1 Fig — The association between DNA variation and family history of cardiac disease (A) and cardiovascular deaths (B). TT, homozygous T-allele carriers; TC, heterozygous carriers; CC, homozygous C-allele carriers. (TIF) [file pone.0164732.s001.tif]
