# Supplementary material for: The Impact of Superoxide Dismutase-1 Genetic Variation on Cardiovascular and All-Cause Mortality in a Prospective Cohort Study: The Yamagata (Takahata) Study
Source: PLoS One. 2016 Oct 18;11(10):e0164732. doi: 10.1371/journal.pone.0164732 (PMC5068777; doi:10.1371/journal.pone.0164732)
Supplement: S1 Text — (DOCX) [file pone.0164732.s002.docx]

**Clinical characteristics related SNP rs17880487.**

As shown in S1 Table, the rs17880487 TT carriers had a lower prevalence of family history of cardiac disease compared to rs17880487 TC and CC carriers. However, there was no significant difference in age, gender, prevalence rates of previous cardiovascular disease, smoking, hypertension, diabetes mellitus, and hyperlipidemia, or biomarkers among the groups. Cardiovascular death was not observed in rs17880487 TT carriers. To assess the impact of rs1041740 TT carriers and rs17880487 TT carriers on the cardiovascular disease, all subjects were divided into subgroups of rs1041740 and rs17880487 genotypes. There were no rs1041740 TT and 17880487 TT, the rs1041740 TT and rs17880487 TC, and the rs1041740 TC and rs17880487 TT carriers. The rs1041740 CC and rs17880487 TT carriers had no family history of cardiovascular disease and cardiovascular deaths (S1 Fig).

**Supplemental table.** Clinical characteristics among rs17880487 genotypes.

| **Variables** | **Homozygous C-allele carriers n = 2305** | **Heterozygous carriers n = 465** | **Homozygous T-allele carriers n = 29** |
| --- | --- | --- | --- |
| **Age, years** | 63 ± 10 | 63 ± 10 | 64 ± 11 |
| **Male/female, n** | 1052/1253 | 204/261 | 14/15 |
| **Family history of cardiovascular disease, n (%)** | 407 (18%) | 69 (15%) | 0 (0%)# |
| **Previous CVD, n (%)** | 323 (14%) | 57 (12%) | 3 (10%) |
| **Previous cancer, n (%)** | 54 (2.3%) | 9 (1.9%) | 1 (3.4%) |
| **Smoking, n (%)** | 728 (32%) | 167 (36%) | 11 (38%) |
| **Hypertension, n (%)** | 844 (37%) | 183 (39%) | 11 (38%) |
| **Diabetes mellitus, n (%)** | 157 (6.8%) | 33 (7.1%) | 1 (3.5%) |
| **Hyperlipidemia, n (%)** | 845 (37%) | 186 (40%) | 13 (44%) |
| **Systolic BP, mmHg** | 134 ± 16 | 134 ± 16 | 135 ± 16 |
| **Diastolic BP, mmHg** | 79 ± 10 | 79 ± 10 | 79 ± 10 |
| **HbA1c, %** | 5.6 ± 0.7 | 5.7 ± 0.7 | 5.8 ± 0.4 |
| **FBG, mg/dL** | 94 ± 17 | 93 ± 16 | 94 ± 16 |
| **Total cholesterol, mg/dL** | 200 ± 31 | 202 ± 32 | 196 ± 38 |
| **HDLc, mg/dL** | 60 ± 14 | 59 ± 14 | 57 ± 16 |
| **Triglyceride, mg/dL** | 105 ± 62 | 112 ± 71 | 117 ± 70 |
| **eGFR, mL/min/1.73 m^2^** | 81 ± 16 | 81 ± 16 | 75 ± 18 |
| **Log_e_ BNP, pg/mL** | 3.02 ± 0.83 | 3.03 ± 0.84 | 2.88 ± 0.92 |
| **Framingham risk score** | 14 ± 4 | 14 ± 4 | 15 ± 4 |
| **All-cause mortality, n (%)** | 167 (7.2%) | 25 (5.4%) | 1 (3.5%) |
| **Cardiovascular mortality, n (%)** | 53 (2.3%) | 4 (0.9%) | 0 (0%) |
| **Non-cardiovascular mortality, n (%)** | 114 (4.9%) | 21 (4.5%) | 1 (3.5%) |

Data are expressed as mean ± standard deviation or number (%)

BNP, brain natriuretic peptide; BP, blood pressure; CVD, cardiovascular disease; eGFR, estimated glomerular filtration rate; FBG, fasting blood glucose; HbA1c, glycosylated hemoglobin A1c; HDLc, high density lipoprotein cholesterol; SNP, single nucleotide polymorphism; SOD, superoxide dismutase. *p<0.05 vs. homozygous C-allele carriers, ^†^p<0.05 vs. heterozygous carriers by analysis of variance (ANOVA) with Bonferroni test. ^#^p < 0.05 by chi-square test.
